# Floodplain farm fields provide novel rearing habitat for Chinook salmon

**DOI:** 10.1371/journal.pone.0177409

**Published:** 2017-06-07

**Authors:** Jacob V. E. Katz, Carson Jeffres, J. Louise Conrad, Ted R. Sommer, Joshua Martinez, Steve Brumbaugh, Nicholas Corline, Peter B. Moyle

**Affiliations:** 1 Center for Watershed Sciences, University of California, Davis, California, United States of America; 2 Department of Wildlife, Fish, and Conservation Biology, University of California, Davis, California, United States of America; 3 California Trout, San Francisco, California, United States of America; 4 Department of Water Resources, Aquatic Ecology Section, Division of Environmental Services, West Sacramento, California, United States of America; Universidade de Aveiro, PORTUGAL

## Abstract

When inundated by floodwaters, river floodplains provide critical habitat for many species of fish and wildlife, but many river valleys have been extensively leveed and floodplain wetlands drained for flood control and agriculture. In the Central Valley of California, USA, where less than 5% of floodplain wetland habitats remain, a critical conservation question is how can farmland occupying the historical floodplains be better managed to improve benefits for native fish and wildlife. In this study fields on the Sacramento River floodplain were intentionally flooded after the autumn rice harvest to determine if they could provide shallow-water rearing habitat for Sacramento River fall-run Chinook salmon (*Oncorhynchus tshawytscha*). Approximately 10,000 juvenile fish (ca. 48 mm, 1.1 g) were reared on two hectares for six weeks (Feb-March) between the fall harvest and spring planting. A subsample of the fish were uniquely tagged to allow tracking of individual growth rates (average 0.76 mm/day) which were among the highest recorded in fresh water in California. Zooplankton sampled from the water column of the fields were compared to fish stomach contents. The primary prey was zooplankton in the order Cladocera, commonly called water fleas. The compatibility, on the same farm fields, of summer crop production and native fish habitat during winter demonstrates that land management combining agriculture with conservation ecology may benefit recovery of native fish species, such as endangered Chinook salmon.

## Introduction

Seasonal inundation of floodplains drive important hydrologic and geomorphic processes that provide substantial trophic benefits to river ecosystems [1, 2]. In California’s Central Valley more than 95% of pre-development floodplain habitats have been leveed and drained, primarily for flood control or conversion to agriculture [3]. Levees alter riverine topography, interrupt natural flow regimes and sever hydrologic, sediment, nutrient and fish connectivity between river channels and adjacent floodplain wetlands [4]. Today, the Central Valley is a patchwork of agricultural lands and communities located on former floodplain wetlands which are now separated from rivers by high, steep levees [5] and only inundate when floodwaters spill into managed floodways or when levees fail during severe storms. As a consequence, access to ancestral floodplain habitats by juvenile salmon and other native fishes has been greatly diminished.

Generally, a species’ abundance is greatest towards the center of its geographic range and declines toward the periphery [6]. At the extreme southern limit of the distribution of Chinook salmon *(Oncorhynchus tshawytscha)*, the Central Valley might, therefore, be expected to be marginal habitat for the species. Instead, the Central Valley was home to one of the largest and most diverse stocks of Chinook salmon [7]. Highly productive and diverse rearing habitats in both freshwater and marine environments are likely part of the explanation for these unexpectedly robust and diverse populations.

Prior to European settlement of the Central Valley, winter and spring floodwaters guided millions of young fall-run Chinook salmon, only a few centimeters long, out of the river channels and onto valley floodplains. Juvenile salmon presumably reared for 1–3 months on productive floodplains, growing rapidly in the process [8]. Declining flows, increases in water temperature and clarity and other cues likely triggered outmigration before floodplains became hydrologically disconnected from stream channels [9]. Sheltered from the current of the main river and supplied with abundant food resources, young salmon that rear on floodplains and other off-channel habitats tend to be larger and in better physical condition that those that rear in the main channel of rivers [10–13]. The extensive photic zone created by large surface areas of shallow floodplain inundation enhances phytoplankton biomass [14–16], zooplankton growth [17, 18], and drift invertebrate biomass [10, 19, 20]. High density of food resources in these shallow, off channel habitats likely contributes to successful foraging and enhanced fish growth. Across many species of anadromous salmonids (e.g., Atlantic salmon *Salmo salar*, steelhead *O*. *mykiss*, Chinook salmon *O*. *tshawytscha*) substantial scientific evidence indicates that body size at ocean entry is an important, if not the primary, indicator of an individual’s probability of returning from the ocean to spawn [21–23]. The rapid growth facilitated by gaining access to floodplain habitats may therefore be critical to conserving self-sustaining populations of Central Valley salmonids [24].

Taken together, the evidence suggests that Central Valley floodplains inundated in midwinter and early spring are a vital habitat link between the upstream gravel beds where salmon spawn and the ocean where they spend the majority of their lives. This study attempted to mimic, on winter-fallowed rice fields, the natural extent and duration of shallow inundation of floodplain habitats that took place in the Central Valley pre-development. The density of invertebrate food resources on which juvenile salmon are foraging in these “managed agricultural floodplains” are similar to those documented on the few relatively natural functioning floodplains left in the Central Valley [11]. Which is to say that the habitats and food densities documented in this study are akin to those under which Central Valley salmon stocks evolved and to which they are adapted. There is therefore, little reason to believe that re-exposing salmon to their ancestral habitat conditions would result in compensatory growth or other related concerns about rapid growth.

Although most Central Valley floodplains have been cut off from river channels by levees, a key feature of regional flood protection is the integration of intentionally inundated flood basins into flood management infrastructure. Known as “bypasses,” these managed floodplains are used to shunt floodwater away from cities and key infrastructure [25]. When the Yolo Bypass (the largest of the Central Valley bypasses) does flood, young salmon successfully use the inundated floodplain for rearing during downstream migration [10, 19, 26]. Dry-season land use within the Yolo Bypass is primarily agricultural and is serviced by extensive irrigation and drainage infrastructure. During winter and spring flooding these bypass farmlands are known to be one of the habitats used by rearing salmon [26]. Use of floodplain habitat by juvenile salmonids in the Central Valley’s current configuration is limited by three major factors: 1) very little inundated floodplain remains, 2) although they constitute the majority of remaining floodplains, managed bypass floodways inundate relatively infrequently, 3) because they are designed and graded to drain rapidly, residency times of floodwaters are shortened on bypasses. Thus when bypasses do flood, fish only have a short time available for floodplain rearing.

Increasingly, Sacramento Valley rice fields are being managed to provide alternative habitat for waterfowl and shorebirds [27–29]. Managed inundation of agricultural fields during the non-growing season has shown that Central Valley rice fields can function as ecological surrogates for lost natural wetland habitats and can aid in the recovery of waterbird populations [30]. This study investigates how intentional winter inundation of agricultural fields on a historical floodplain might be used to benefit native fish. Specifically, our study was designed to test if post-harvest rice fields flooded from irrigation canals during the winter non-growing season could function as rearing areas for juvenile Chinook salmon. We tested this hypothesis by rearing juvenile fall run Chinook salmon (a federal species of special concern) in a post-harvest rice field on the Yolo Bypass near Sacramento, California in February and March of 2012. Environmental conditions proved sufficient for fish survival as evidenced by rapid growth and robust body condition.

## Methods

### Study location

At 24,000 hectares, the Yolo Bypass is the Central Valley’s largest contiguous floodplain still regularly inundated by floodwaters [19]. Flooding occurs in two out of three years on average, typically between the months of December and April but flood events vary from several days to months in duration. As a result, the bypass represents one of the most frequent large-scale connections of river and floodplain left in the Central Valley. The seasonal wetlands of the Yolo Bypass provide critical rearing habitat for juvenile Chinook salmon [10] and are a vital component of the Pacific Flyway, a migration pathway used by 20% of North America’s waterfowl [31].

Located approximately 8 km west of the city of Sacramento, the Yolo Bypass occupies a portion of the historical flood basin in the region. The current configuration is a partially leveed basin that is seasonally inundated from the Sacramento River via simple weirs and local tributaries. It functions to prevent damaging floods by bypassing high flows around the Sacramento Metropolitan Area, thereby relieving pressure on urban levees during high flow events. The bypass area is covered by floodway easements held by the State of California, making all other land uses subservient to flood control. A major land use in the Yolo Bypass is agriculture, with rice the primary crop. Additionally, wild rice, tomatoes, corn, safflower and melons are grown and substantial areas are managed as irrigated pasture or kept fallow. Extensive areas within the bypass are also managed for waterfowl habitat and hunting.

**Fig 1:**
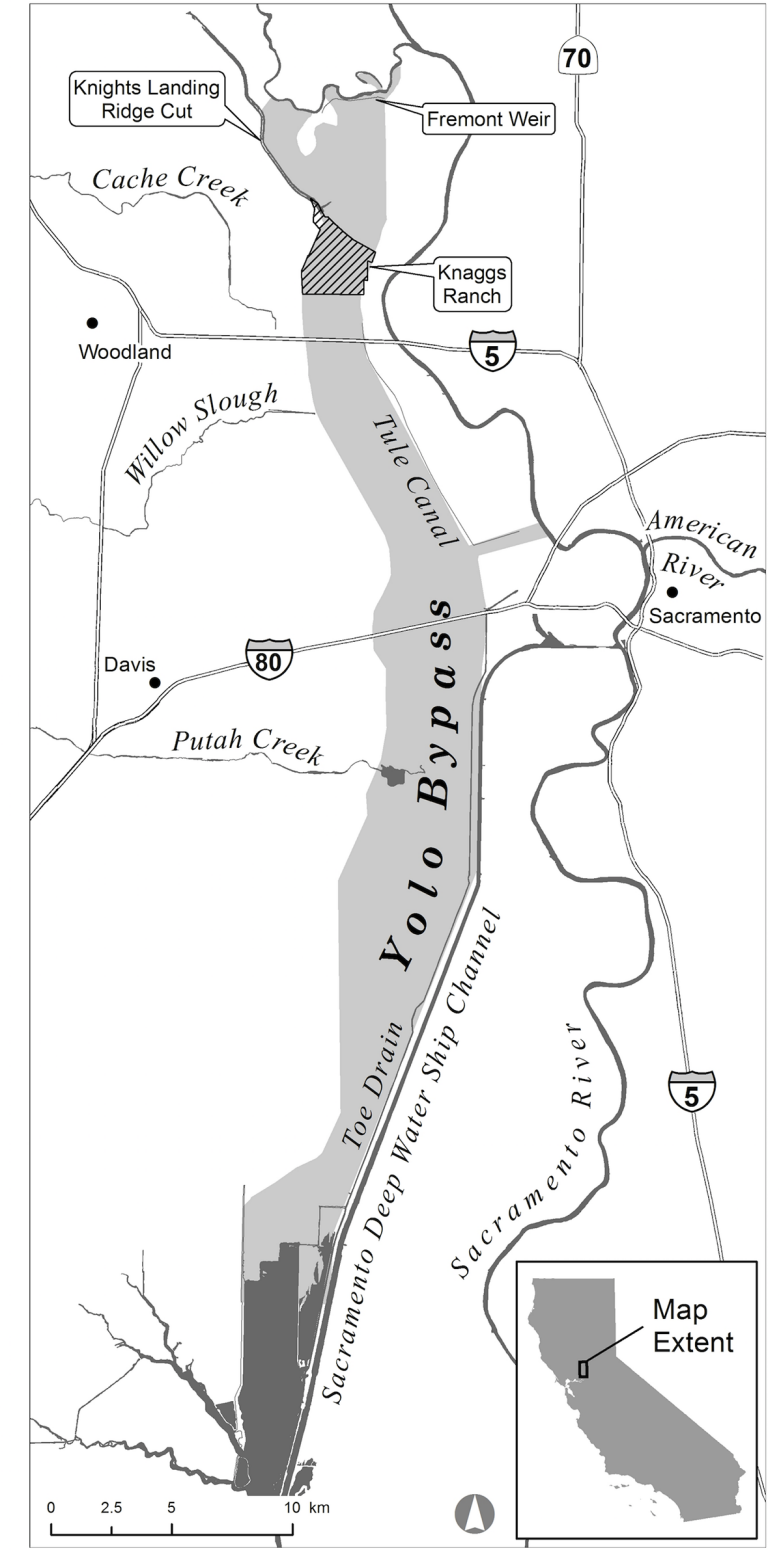
Location of Yolo Bypass and important landmarks including Knaggs Ranch.

The study was located on the Knaggs Ranch (38.698431° N, -121.658506° W; Fig 1), an agricultural parcel in the northern Yolo Bypass with a total acreage of 670 hectares. All studies were done with the knowledge and cooperation of the landowners. A drainage canal called the Knights Landing Ridge Cut enters the Knaggs property at its northwest corner. This canal was built early in the 20^th^ century to direct floodwaters in the Colusa Basin to flow into the Yolo Bypass. Currently 636 hectares of the ranch are farmed to rice and irrigated with water from the Knights Landing Ridge Cut, supplemented with groundwater from on-site wells.

The study year 2012 was relatively dry with no Sacramento River overflow into Yolo Bypass. This provided an opportunity to examine the potential for managed inundation of agricultural lands to improve rearing conditions for young salmon. Previous studies have reported on the successful use by juvenile salmon of Central Valley floodplain habitats inundated during natural flood events [10, 11]. We hypothesized that farm fields intentionally inundated during winter using water from irrigation canals could provide similar aquatic habits to natural floodplains where salmon growth, condition, and survival would all be relatively high. Second, we predicted that different agricultural habitat types in the field would result in differential growth, condition, or survival.

**Fig 2:**
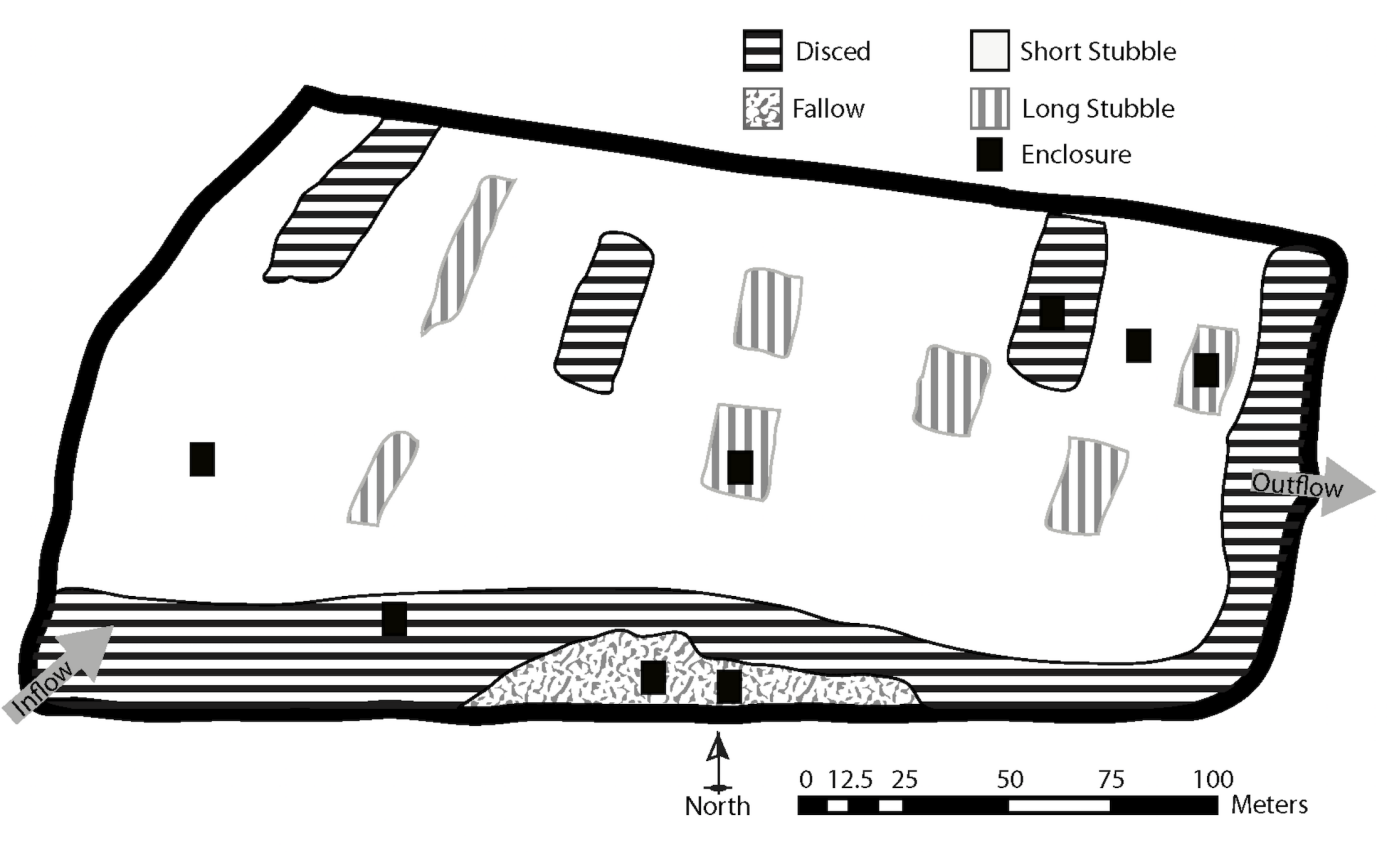
Map of agricultural substrates in the approximately two-hectare experimental field. Shading represents different agricultural substrates. Background matrix (white) was short stubble. Arrows represent direction of water flow.

The two ha field (Fig 2) contained four substrate types: newly ploughed soil (disced), rice stubble cut to an average of 5 cm (low stubble), rice stubble cut to a length of 35 cm (long stubble), and annual herbaceous vegetation (fallow). Two 3 x 4.5m enclosures were placed on each substrate type (eight total) so that fish could be recaptured in order to compare substrate-specific growth rates. Enclosures were walled with 1.2 m high extruded plastic fencing (3 mm opening mesh) trenched into the soil and open to the sky. Enclosures were haphazardly placed along a depth gradient in the field from 23 cm to 69 cm (Fig 2). Water was gravity fed into the field at its southwestern corner and drained on its eastern side. Both inlet and outlet were screened with the same material as the enclosures. Flow rates fluctuated according to water elevation in the irrigation supply canal, ranging from 0 to 0.08 cubic meters per second. During the course of the experiment, fish could have escaped on three occasions: once erosion caused a small opening beneath the inlet fish screen and twice waves generated by high winds breached small portions of the levee.

Water temperatures were recorded at 10-minute intervals using Onset HOBO loggers (Bourne, Mass., USA) installed within the field directly adjacent to the inlet and outlet. Dissolved oxygen, electrical conductivity, pH and turbidity were measured with a YSI multi-parameter sonde every 1–3 days.

### Source and sampling of Chinook salmon

Juvenile Chinook salmon were reared in winter and early spring in a post-harvest 2-hectare rice field (Fig 2). Approximately 10,218 juvenile fish, averaging 48 mm and 1.1 g (n = 50), were planted into the inundated field on January 31, 2012 for an initial density of 5,000 fish/hectare (0.5 fish/m^2^). These densities were comparable to prior observations of wild fish during natural flood events in Yolo Bypass [26]. Fish were removed on March 12, 2012. All fish were obtained from the Feather River Hatchery, where their adipose fins were clipped and tagged using half-length decimal coded-wire tags (Northwest Marine Technology, Inc., Washington) to identify them as a unique group. Fish were transported to the experimental site using a fish transport truck equipped with aerators.

Two hundred and ninety-nine fish were implanted with 8 mm passive integrated transponder (PIT) radio frequency identification tags (Biomark, Boise Idaho, USA) at the field site, allowing us to track performance of individual fish. One hundred thirty-nine PIT-tagged fish were released into the flooded field to swim freely (mean fork length 48.7 ± 0.2mm and weight 1.10 ± 0.01 g), while the remaining 160 PIT-tagged fish were placed into the eight enclosures (mean fork length 47.7 ± 0.2mm and weight 1.10 ± 0.07 g)–two enclosures per habitat type, 20 fish per enclosure. Enclosure stocking density was 1.5 fish/m^2^.

Enclosures allowed reliable recapture of individual salmon in order to track and compare individual growth rates across depth and substrate. Parallel biweekly measurements of free-swimming PIT-tagged fish captured via beach seine provided an experimental control to examine whether the effects of rearing in enclosures (foraging efficiency, stocking density, etc.) substantially altered growth rates. Fish in enclosures were recaptured every two weeks for the six-week duration of the study using Seine nets. After 42 days, the field was drained into a perennial channel that connects to the Sacramento River (Fig 1). Fish were captured in a mesh trap and counted as they exited the field. A subsample of 50 fish without PIT tags and all PIT-tagged fish were measured for fork length (mm ± 1) and weighed using an Ohaus Scout Pro field balance (g ± 0.01). Weights were taken with the scale placed on level ground in a closed clear plastic box to minimize measurement error caused by wind or motion.

### Zooplankton samples

To assess the zooplankton community in the experimental field, zooplankton net tows were conducted at four randomly assigned locations within the field. At each location a 30 cm diameter, 153 μm zooplankton net attached to five meters of rope was thrown the full five-meter distance and retrieved four times. One throw in each of the cardinal directions. Zooplankton density was calculated as the number of individuals per cubic meter of water sampled where sample water volumes are equal the area of the mouth of the net multiplied by the distance towed. Sampling was performed on February 14 and 27. All samples were preserved in a solution of 95% EtOH. Due to the density of crustaceans within the zooplankton samples sub-sampling was employed. Samples were rinsed through a 150 μm mesh and then emptied into a beaker. The beaker was filled to the desired volume, depending on the number of individuals within the sample, and then sub-sampled with a 1 mL large-bore pipette. If densities were still too great for enumeration, the sample was divided using a Folsom zooplankton splitter. The volume and number of aliquots removed was recorded and used to obtain total estimates of zooplankton. Zooplankton samples were sorted until more than 300 individuals were counted.

Zooplankton were enumerated and identified with the aid of a dissecting microscope at four times magnification. Zooplankton were identified to order or family using keys from pertinent ecological literature [32, 33].

### Stomach contents

At the end of the study period, three fish per enclosure and 10 free-swimming fish were collected for diet analysis. Fish were euthanized in the field via directed concussive impact to cranial foci (as per UC Davis animal care protocol #17137) and immediately placed on ice to be utilized for subsequent stomach content analysis. Collected samples were kept in a freezer at UC Davis at -10°C until being thawed for analysis, at which time stomachs were removed and the contents were emptied into water filled slide trays. The contents were identified and enumerated as explained in the zooplankton methods section above.

### Statistical analysis

Apparent growth rate of free-swimming (non PIT-tagged) fish was calculated as the difference of sample means for both lengths and weights taken on the day fish were planted (n = 50) and when the field was drained (n = 98). Individually marked (PIT tagged) fish were analyzed by a repeated measures analysis. Post hoc comparisons were performed using the Tukey-Kramer method for multiple testing. Statistical significance was declared at the 0.05 level. All statistical analysis was done in JMP pro v. 10.0.2 (SAS Institute Inc.). Relation of length to weight is reported as Fulton’s index of body condition (K), calculated as 100,000 times weight in grams divided by the cube of fork length in mm [34].

### Ethics statement

All necessary permits were obtained for the described field collections and experiments (California Department of Fish and Game Scientific Collecting permit # SC-12677). This study was carried out in accordance with the protocol approved by the Institutional Animal Care and Use Committee of the University of California at Davis (permit #17137).

## Results

### Physical conditions

**Fig 3:**
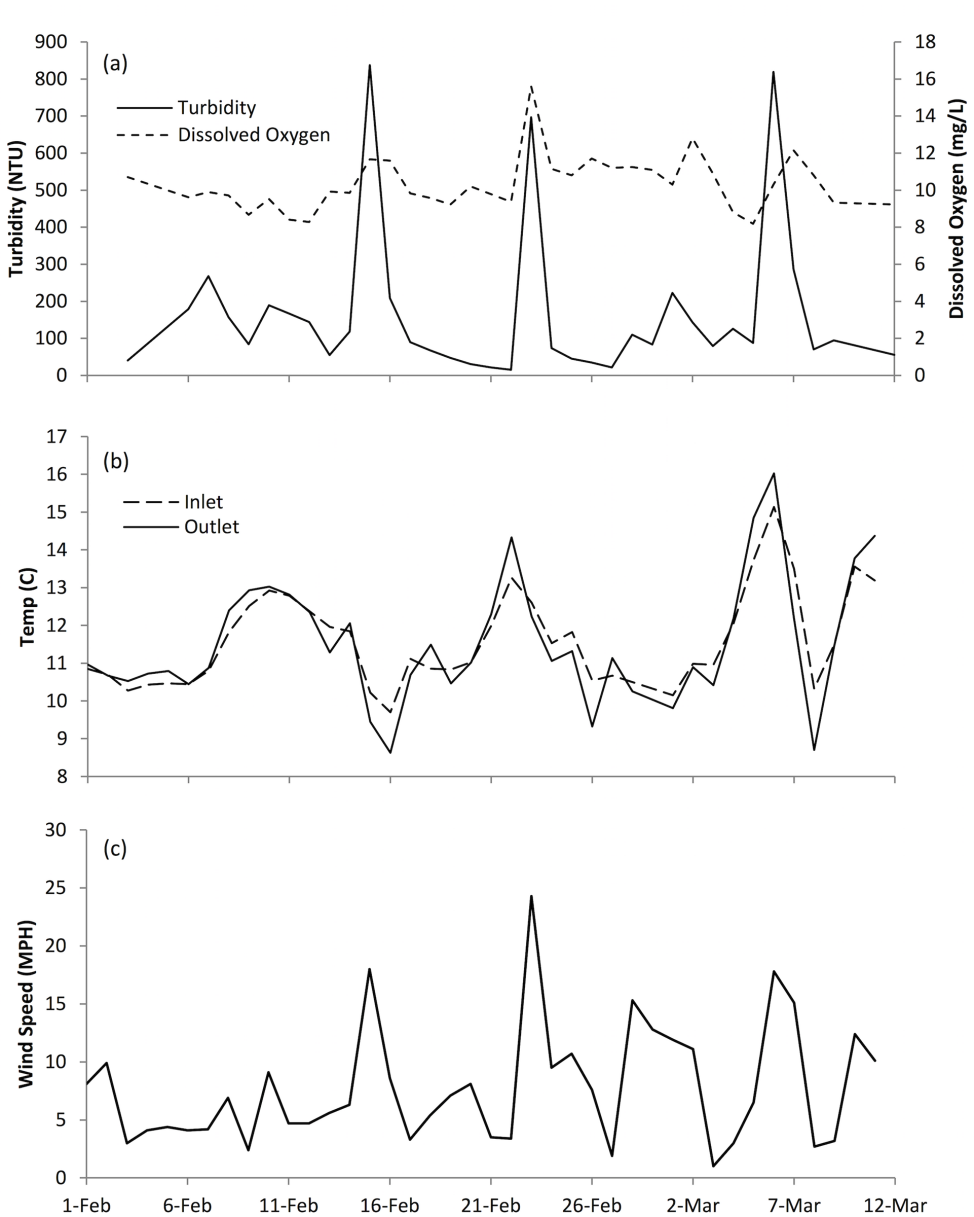
Water quality and wind speeds for the duration of the 2012 study. (a) Turbidity and Dissolved Oxygen, (b) Inlet and Outlet Temperatures, and (c) Wind Speed.

Water temperatures were highly variable. This was expected based on the large area of shallow, low-velocity habitat. During certain periods, the outlet was colder than the inlet by as much as 1–2°C, likely due to evaporative cooling from periodic high winds (Fig 3). Temperatures during the study were within suitable limits for Chinook salmon [35].

Turbidity remained high and variable throughout the study period, likely due to fine sediments remaining in suspension due to water turbulence generated by wind. Readings near the outlet of the field ranged from as low as 30 to as high as 837 NTU during short-term spikes (Fig 3). Dissolved oxygen remained high throughout the study with occasional spikes, also likely attributable to mixing driven by strong winds (Fig 3).

### Zooplankton

**Fig 4:**
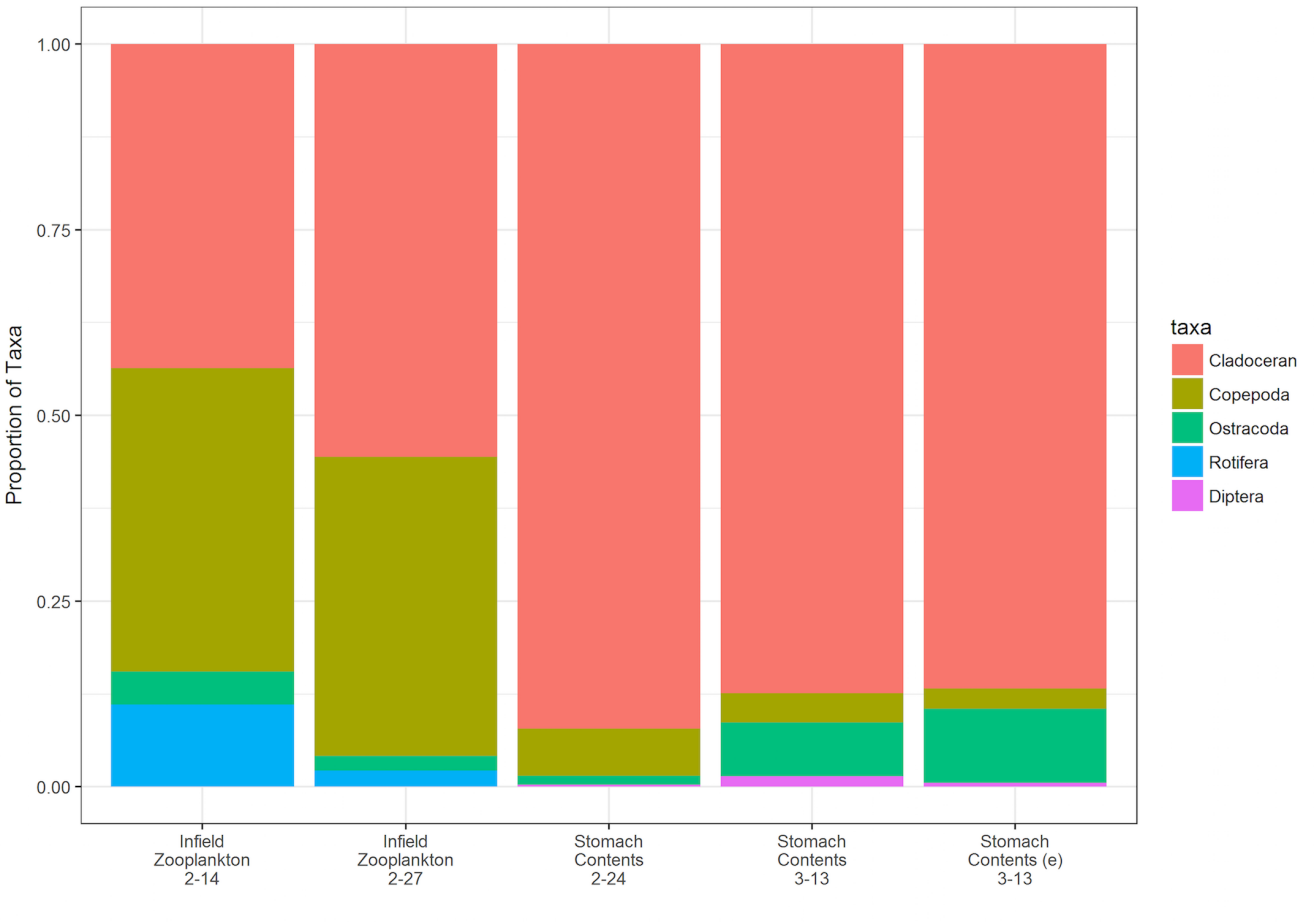
Proportion of zooplankton taxa found in (from left to right) the water column of the flooded rice field on February 14^th^ (n = 4), Feb 27^th^ (n = 4), the stomach contents of free-swimming juvenile Chinook salmon collected on Feb. 24^th^ (n = 6) and March 13^th^ (n = 19) and the stomach contents of enclosure-reared (e) juvenile Chinook salmon collected on March 13^th^ (n = 48).

Cladocerans were the most abundant taxa in the zooplankton community during both sample periods (Fig 4) with densities of 4,511 individuals/m^3^ on February 14^th^ and 4,150 ind./m^3^ on February 27^th^ (n = 4 per sampling period). Copepods (including both copepodids and adults) were the second most abundant taxa with densities of 4,017 ind./m^3^ during the first sample period and 1,903 ind./m^3^ during the second. Ostracods densities dropped between the two sample periods from 1,603 ind./m^3^ to 311 ind./m^3^. Rotifers densities ranged from 1,147 ind./m^3^ on the 14^th^ of February to 163 ind./m^3^ on February 27^th^.

### Stomach contents

Cladocerans comprised 92% of stomach contents of free-swimming fish sampled on Feb. 24^th^ (n = 6), 90% for free-swimming fish (n = 29) collected on March 13^th^ and 87% for enclosure-reared fish (n = 48) collected on March 13^th^ (Fig 4). Ostracods, copepods and dipterans (in descending order of abundance) compromised the remaining proportion of taxa found in salmon stomachs (Fig 4).

### Growth of Free-swimming fish

Upon completion of the 42-day experiment, the mean length and weight of free-swimming PIT-tagged fish were 78.0 ± 0.5 mm (all variances expressed as standard error) and 5.74 ± 0.11 g, respectively (n = 50). These values represent mean growth rates of 0.70 ± 0.01 mm/d and 0.11 ± 0.01 g/d. Fulton’s condition factor was 1.21 ± 0.01. An unknown number of fish escaped. Consequently, estimates of survival during the study period could not be derived. Of the approximately 10,218 fish stocked into the study area, 5,835 (~57%) were recovered.

Apparent growth of free-swimming fish (not PIT-tagged) was 0.76 ± 0.01 mm/d (n = 50).

### Growth of enclosure-reared fish

**Fig 5:**
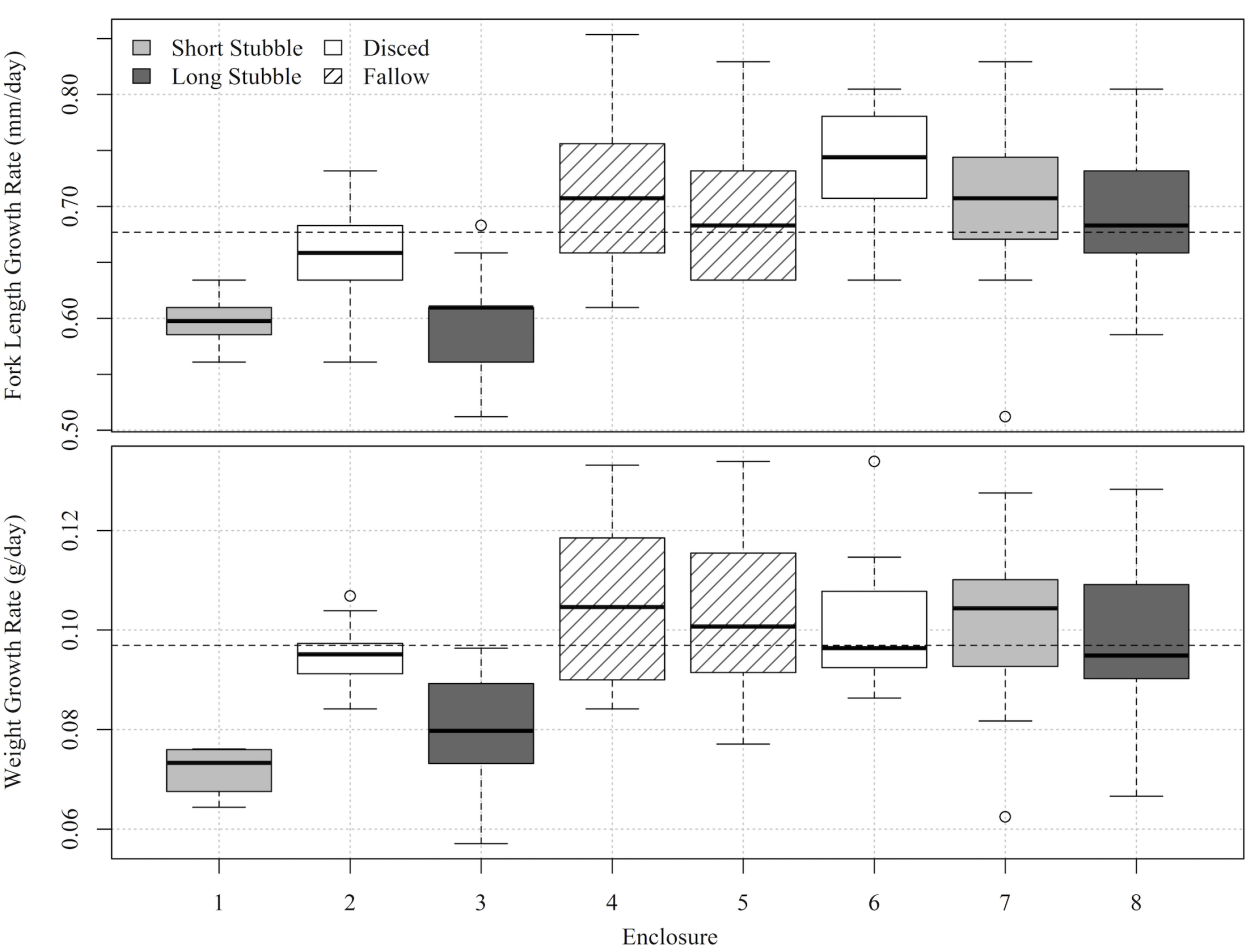
Growth rates (mm/d, g/d) of juvenile Chinook salmon across the experimental enclosures. Dotted line represents mean value for all enclosures.

One hundred and thirteen fish were recovered from enclosures but weight data was corrupted by scale malfunction in two instances so that only 111 could be used in weight calculations. Mean length and weight of enclosure-reared fish at study’s end were 75 mm ± 0.3 (n = 113), and 5.05 ± 0.07 g (n = 111), respectively, representing mean growth rates of 0.68 ± 0.01 mm/d (n = 113), and 0.10 ± 0.00 g/d (n = 111). Growth rates varied significantly between enclosures (length, P < 0.01; weight, P < 0.01) and substrate treatment (length, P < 0.01; weight, P < 0.01). Tukey post hoc revealed these effects to be driven by poor growth performance in enclosures one (low stubble) and three (long stubble) (Fig 5). Mean Fulton’s condition factor was 1.18 ± 0.01 and was not significantly different across substrates (P = 0.09) or by enclosure (P = 0.12).

## Discussion

Prior to development, Central Valley floodplains were regularly inundated multiple times a year and stayed wet for prolonged periods. In contrast, modern bypass floodplains are designed to inundate only during large flood events and are engineered and graded for rapid drainage, dramatically reducing inundation duration compared to natural conditions. Managed inundation of winter fields appears to mimic the natural prolonged residence times and hydrologic patterns under which Central Valley salmon stocks evolved and to which they are adapted. Creation of an artificial flood on a managed agricultural floodplain appears to have supported a robust aquatic food web and provided floodplain habitat conditions conducive to rapid growth of juvenile salmon.

While this study demonstrates the potential to reconcile management of agricultural working landscapes with recovery of Chinook salmon populations, the results do not detract from the need to restore suitable natural (i.e., non-agricultural) off-channel salmon habitats wherever feasible to maintain life history diversity. Considerably more work is needed to develop specific management strategies that increase floodplain benefits to juvenile Chinook salmon. Examples of issues to be resolved in the future include: 1) improving salmon floodplain access by increasing connectivity between floodplains and rivers; 2) evaluating use of water retention infrastructure to extend the duration of flood events; 3) determining habitat types that maximize food web production, salmon growth, and survival; 4) determining whether some managed conditions could create adverse water effects or enhance predation; and 5) determining how all four Central Valley runs of wild Chinook salmon, steelhead *(O*. *mykiss*) and native cyprinid minnows can benefit from floodplain management.

The great preponderance of growth studies of juvenile salmon in California have been conducted on fish reared within the river channel (S1 Table). While the growth of juvenile Chinook salmon in this study are among the most rapid ever recorded in the Central Valley (see S1 Table for comparisons among other studies), they are similar to the few other studies that have documented floodplain-specific growth. The mean growth rate of free-swimming fish in our study (0.70 ± 0.01 mm/d) falls in between those recorded during natural flooding events on the Yolo Bypass in 1998 (0.80+/-0.06 mm/d) and 1999 (0.55+/-0.06 mm/d). However, because these studies relied on recapture of individuals downriver in the San Francisco Estuary at Chipps Island, the relative contribution to growth from floodplain and estuarine habitats is not known [10]. Penned juvenile Chinook salmon in our study grew at 0.68± 0.01 mm/d—28% faster than those documented by using similar caging methods on complex natural habitats of the Cosumnes River floodplain during a natural flood event [11].

Salmon in this study fed primarily on zooplankton, namely cladocerans. The salmon had a higher relative abundance of cladocerans in their stomachs (92%) than was found in the zooplankton community in the inundated rice field (52%). Similar results of selective feeding on large bodied cladoceran species have been found for Chinook salmon in other studies where salmon entered low gradient lentic environments [36–38]. The cladoceran community in the experimental field was dominated by large bodied *Simocephalus spp*. The abundance, large size, and slow movement of cladocerans may have led to more efficient prey capture than for other potential prey species such as chironomid midges [39, 40]. Floodplain inundation recruits terrestrial plant material into the aquatic environment, increases average daily temperatures and expands the photic zone, likely creating conditions conducive to increased phytoplankton biomass with resultant increases in zooplankton population growth rates, abundance and densities.

Hatchery fish were used in this study because of the difficulty of obtaining permits for naturally spawned fish. However, increased growth rates are likely to characterize all juvenile salmonids, irrespective of hatchery or in-river origin, that gain access to the abundant food resources on inundated floodplains [41].

**Fig 6:**
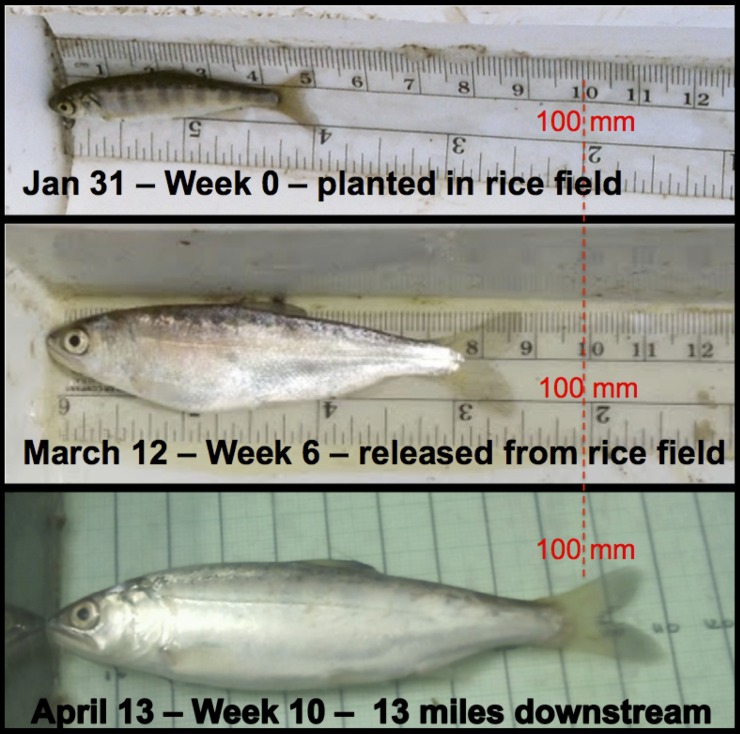
Representative juvenile Chinook salmon before (top) and after (middle) rearing for six weeks on the Knaggs Ranch experimental agricultural floodplain on Yolo Bypass. Bottom picture is of a tagged Knaggs fish incidentally recaptured in a rotary screw trap in the Yolo Bypass Toe Drain 13 miles downstream of the release site four weeks after the termination of the experiment.

The relatively large size and good body condition of floodplain-reared out-migrants (Fig 6 and see Supporting Information for comparisons among other studies) is likely to be particularly important because smaller individuals are more vulnerable to predation and other causes of size-dependent mortality [42]. Accumulated fat reserves resulting from floodplain rearing may increase survival by buffering effects of subsequent poor foraging conditions encountered during outmigration or upon arrival in the marine environment. Lack of access to floodplain habitats may also constrain resilience of Central Valley salmon stocks by condensing the range of outmigration timing [43].

Rearing on Yolo Bypass provides an additional benefit by routing fish into the relatively high quality habitat in the Northwest Delta and away from migration pathways into the central Delta where mortality rates increase significantly due to higher predation, poor water quality, and the possibility of entrainment in large water export pumps [44]. Floodplains are therefore an important piece of the spatially and temporally diverse mosaic of riverine habitats needed to facilitate the full range of life-history expression on which resilient, self-sustaining populations of salmon depend.

## Conclusions

Previous studies in the Central Valley found that rearing in complex off-channel habitats during natural inundation resulted in rapid growth of juvenile Chinook salmon [10, 11, 13]. However, very little of this type of habitat remains accessible to juvenile salmon.

Juvenile Chinook salmon given access to Yolo Bypass farm fields managed as winter floodplains grew at rates similar to those measured under natural flood conditions [10]. The overall rapid growth and robust body condition of the salmon in this study demonstrates that winter flooding of rice fields during the agricultural non-growing season can provide high quality habitat for rearing juvenile Chinook salmon. These results suggest that changes to agricultural management and infrastructure that increase the frequency and extend the inundation duration of bypass flood events could allow floodplain farm fields to serve as large-scale surrogates for floodplain wetlands, which once were important salmon-rearing habitat.

This study also demonstrates the potential of managing a working agricultural landscape for the combined benefits to fisheries, farming, flood protection, and native fish and wildlife species [19, 27, 28]. This relatively balanced outcome allows native species to exploit working agricultural lands as high-value habitat, thereby reconciling multiple resource management and wildlife objectives. These results should have broad applicability for the management of floodplains throughout California and beyond.

## Supporting information

S1 Table Available growth and condition factors for out-migrating Chinook salmon between 65–100 mm fork length in the Sacramento Valley, Sacramento-San Joaquin Delta, San Francisco Estuary, and California coastal ocean. (DOCX)

S1 Dataset Fish growth, zooplankton abundance and stomach contents data from experiments on Knaggs Ranch in Yolo Bypass, California. 2012.
(XLS)
