# Supplementary material for: Floodplain farm fields provide novel rearing habitat for Chinook salmon
Source: PLoS One. 2017 Jun 7;12(6):e0177409. doi: 10.1371/journal.pone.0177409 (PMC5462374; doi:10.1371/journal.pone.0177409)
Supplement: S1 Table — (DOCX) [file pone.0177409.s001.docx]

**Supporting Information**

**S1 Table 1. Available growth and condition factors for out-migrating Chinook salmon between 65-100mm fork length in the Sacramento Valley, Sacramento San Joaquin Delta, San Francisco Estuary, and California coastal ocean.**

| **Sampling**  **Location** | **Author(s) or**  **data collector(s)** | **Year**  **of data** | **Growth rate**  **(mm/d)** | **Apparent growth rate (mm/d)** | **Stan.**  **dev.** | **Stan.**  **error** | **Condition**  **Factor**  **(K)** | **n** | **Sampling method** |
| --- | --- | --- | --- | --- | --- | --- | --- | --- | --- |
| Upper Sac | Kjelson et al. 1982 | 1981 |  | 0.33 |  |  |  |  | Seine |
| Delta | Kjelson et al. 1982 | 1980 |  | 0.86 |  |  |  |  | Seine |
| Delta | Kjelson et al. 1982 | 1981 |  | 0.53 |  |  |  |  | Seine |
| Gulf of Farallones | R. B. MacFarlane et al. 2005 | 1998 |  | 0.85 | 0.13 |  |  |  | Trawl/ otolith increment analysis of growth rates |
| Gulf of Farallones | R. B. MacFarlane et al. 2005 | 1999 |  | 0.55 | 0.06 |  |  |  | Trawl/ otolith increment analysis of growth rates |
| Lower Sac, Delta | Sommer et al. 2001 | 1998 |  | 0.52 | 0.18 | 0.02 |  | 10 | Trawl |
| Yolo Bypass, Delta | Sommer et al. 2001 | 1998 |  | 0.80 |  | 0.06 |  | 9 | Trawl |
| Lower Sac, Delta | Sommer et al. 2001 | 1999 |  | 0.43 | 0.08 | 0.03 |  | 8 | Trawl |
| Yolo Bypass, Delta | Somer et al. 2001 | 1999 |  | 0.55 |  | 0.06 |  | 9 | Trawl |
| Yolo Bypass | Sommer (unpublished data) | 1999 |  | 0.38 | 0.60 |  |  | 40 | Rotary Screw Trap (RST) |
| Yolo Bypass | Sommer (unpublished data) | 2000 |  | 0.55 | 0.19 |  |  | 45 | RST |
| Yolo Bypass | Sommer (unpublished data) | 2004 |  | 0.67 | 0.12 |  |  | 25 | RST |
| **Yolo Bypass** | **This study** | **2012** | **0.70** |  | **0.09** | **0.01** | **1.21** | **50** | **PIT free swimming** |
| **Yolo Bypass** | **This study** | **2012** | **0.68** |  | **0.08** | **0.01** | **1.18** | **107** | **PIT enclosures** |
| **Yolo Bypass** | **This study** | **2012** |  | **0.76** | **0.11** | **0.01** |  | **98** | **Free swimming (not PIT tagged)** |
| Butte Creek &  Sutter Bypass | McReynolds, Ward, Garman | 1999-00 |  | 0.66 |  |  |  |  | RST |
| Butte Creek &  Sutter Bypass | Ward, McReynolds, Garman | 2000-01 |  | 0.57 |  |  |  |  | RST |
| Cosumnes River & floodplain | Jeffres et al. 2008 | 2004-05 |  | 0.16-0.51 |  |  |  |  | Enclosures |
| Deer Creek | Matt Johnson- DFG | 1995 |  |  |  |  | 1.02 | 187 | RST |
| Deer Creek | Matt Johnson- DFG | 1996 |  |  |  |  | 1.09 | 126 | RST |
| Deer Creek | Matt Johnson- DFG | 1997 |  |  |  |  | 1.05 | 74 | RST |
| Deer Creek | Matt Johnson- DFG | 1999 |  |  |  |  | 1.06 | 118 | RST |
| Deer Creek | Matt Johnson- DFG | 2000 |  |  |  |  | 1.08 | 148 | RST |
| Deer Creek | Matt Johnson- DFG | 2001 |  |  |  |  | 1.09 | 528 | RST |
| Deer Creek | Matt Johnson- DFG | 2002 |  |  |  |  | 1.1 | 566 | RST |
| Deer Creek | Matt Johnson- DFG | 2003 |  |  |  |  | 1.11 | 186 | RST |
| Deer Creek | Matt Johnson- DFG | 2004 |  |  |  |  | 1.11 | 282 | RST |
| Deer Creek | Matt Johnson- DFG | 2005 |  |  |  |  | 1.1 | 746 | RST |
| Deer Creek | Matt Johnson- DFG | 2006 |  |  |  |  | 1.11 | 35 | RST |
| Mill Creek | Matt Johnson- DFG | 1996 |  |  |  |  | 1.07 | 9 | RST |
| Mill Creek | Matt Johnson- DFG | 2000 |  |  |  |  | 1.15 | 54 | RST |
| Mill Creek | Matt Johnson- DFG | 2001 |  |  |  |  | 1.18 | 391 | RST |
| Mill Creek | Matt Johnson- DFG | 2002 |  |  |  |  | 1.14 | 695 | RST |
| Mill Creek | Matt Johnson- DFG | 2003 |  |  |  |  | 1.13 | 98 | RST |
| Mill Creek | Matt Johnson- DFG | 2004 |  |  |  |  | 1.11 | 137 | RST |
| Mill Creek | Matt Johnson- DFG | 2005 |  |  |  |  | 1.15 | 356 | RST |
| Mill Creek | Matt Johnson- DFG | 2006 |  |  |  |  | 1.18 | 77 | RST |
| Yuba River | D. Massa- Yuba River Management Team | 1999-2000 |  |  |  |  | 0.91 |  | RST |
| Yuba River | D. Massa- Yuba RMT | 2000-2001 |  |  |  |  | 0.88 |  | RST |
| Yuba River | D. Massa- Yuba RMT | 2001-2002 |  |  |  |  | 0.81 |  | RST |
| Yuba River | D. Massa- Yuba RMT | 2003-2004 |  |  |  |  | 0.89 |  | RST |
| Yuba River | D. Massa- Yuba RMT | 2004-2005 |  |  |  |  | 0.84 |  | RST |
| Yuba River | D. Massa- Yuba RMT | 2005-2006 |  |  |  |  | 0.85 |  | RST |
| Yuba River | D. Massa- Yuba RMT | 2006-2007 |  |  |  |  | 0.85 |  | RST |
| Yuba River | D. Massa- Yuba RMT | 2007-2008 |  |  |  |  | 0.90 |  | RST |
| Yuba River | D. Massa- Yuba RMT | 2008-2009 |  |  |  |  | 0.84 |  | RST |
| Coleman hatchery | Bob Null, Arnold Ammann, Steve Zeug, Jason Hassrick, Bill Becket, Gabe Singer | May-2012 |  |  |  |  | 1.10 | 119 | in hatchery |
| Lower American American River | Snider & Titus | Jan-July 1994 |  |  |  |  | 1.14 |  | RST |
